# Analysis of circulating insulin-like growth factor-1 (IGF-1) and IGF binding protein-3 (IGFBP-3) in tobacco smokers and non-smokers

**DOI:** 10.1186/1617-9625-1-2-157

**Published:** 2002-06-15

**Authors:** RM Palmer, RF Wilson, PY Coward, DA Scott

**Affiliations:** 1 Department of Periodontology and Preventive Dentistry, King's College London, London, UK; 2 Dental Clinical Research, King's College London, UK; 3 Department of Oral Biology, University of Manitoba, Canada

## Abstract

**Background:**

IGF-1 and the major serum IGF-1 binding protein, IGFBP-3, are under extensive investigation as potential prognostic markers of specific malignancies and vascular diseases. However, there is conflicting evidence that tobacco smoking may influence systemic concentrations of IGF-1 and IGFBP-3.

**Subjects and methods:**

Serum concentrations of IGF-1 and IGFBP-3 were measured in 20 smokers and 20 non-smokers, matched for age and gender. Serum concentrations of cotinine, the major metabolite of nicotine, and ICAM-1, known to exhibit a dose-dependent relationship with cotinine, were also assayed.

**Results:**

There was no difference between the systemic concentrations of IGF-1 or IGFBP-3 found in smokers and non-smokers (IGF-1: mean [s.d]; 104 [29] vs 101 [24] ng ml^-1^, respectively; and IGFBP-3: 2562 [522] vs 2447 [570] ng ml^-1^, respectively). Similarly, there was no correlation between serum cotinine and IGF-1 or IGFBP-3 concentrations in smokers. Soluble ICAM-1 concentrations were significantly increased in smokers, compared to non-smokers (mean [s.d]; 258 [60] vs 194 [50] ng ml^-1^, respectively; p = 0.002).

**Conclusion:**

There was no relationship noted between tobacco smoking and either IGF-1 or IGFBP-3. These data suggest that smoking would not appear to be a major confounder of the reported clinical associations between IGF-1, IGFBP-3, or IGF-1/IGFBP-3 ratios and specific disease entities.

## Introduction

Insulin-like growth factor (IGF-1) is a 70 amino acid (7.6 kDa) cytokine constitutively produced by the liver due to stimulation by insulin [1] and growth hormone [2]. IGF-1, which exhibits a high degree of structural homology to proinsulin, acts to lower glucose levels [3,4] and suppress insulin production [5].

There are at least six IGF binding proteins (IGFBPs). IGFBP-3 is the major IGFBP in serum, with more than 90% of circulating IGF-1 bound in a ternary complex with IGFBP-3 and acid-labile subunit, and only 1% of the total serum IGF-1 normally circulates in free form [4-8]. While IGFBP-3 is expressed in many tissues, non-parenchymal liver cells are considered the primary source of circulating IGFBP-3. IGFBP-3 is susceptible to cleavage by a variety of proteases, including cathepsin G [9], neutrophil elastase [9], and metalloproteases [10,11]. Proteolytic cleavage of systemic IGFBP-3 would increase the amount of free IGF-1, and be expected to alter the systemic IGF-1/IGFBP-3 ratio.

IGF-1 and IGFBP-3 both promote the differentiation of bone, with systemic levels of IGF-1 and IGFBP-3 positively associated with bone mass density [12]. Indeed, there is increasing evidence that alterations to the IGF-1/IGFBP axis are associated with osteoporosis [13-15]. IGF-1 has been shown to act as a potent mitogen, with anti-apoptotic actions, on various cancer cells [16], whereas IGFBP-3 may have IGF-1-independent pro-apoptotic activity [17,18]. An aetiological role for IGF-1 and IGFBP-3 in malignancy is supported by clinical studies that associate high IGF-1 and low IGFBP-3 levels with increased risk of several cancers, such as breast, colorectum, and lung cancer [19-21]. Dysregulation of the IGF/IGFBP system is also thought to influence the development of coronary atherosclerosis and other vascular diseases [22-26].

It should be noted that specific malignancies (including colorectal, lung and breast cancers), osteoporosis, and vascular disease are all tobacco-induced and/or – exacerbated diseases. Previous studies have come to conflicting conclusions on a potential relationship between IGF-1, IGFBP-3 and smoking status [20,21,23,27-30]. Considering the important potential of the IGF/IGFBP system as prognostic markers of malignancy and vascular disease, there is a pressing need to clarify the relationship between tobacco use and the systemic load of IGF-1 and the major IGF-1 binding protein, IGFBP-3. Age and gender have been shown to correlate negatively with serum IGF-1 and IGFBP-3 concentrations [12,20,23,27,31]. We have previously shown that circulating concentrations of soluble intercellular adhesion molecule-1 (sICAM-1; CD54) are known to be significantly elevated in smokers, in a dose-dependent manner, making this a suitable positive control serum marker [32,33]. Therefore, we examined the circulating concentrations of IGF-1, IGFBP-3, and soluble ICAM-1 in 20 smokers and 20 non-smokers, matched for age and gender, and whose smoking status was confirmed by serum cotinine analysis.

## Subjects, Materials, and Methods

### Subjects

Serum was obtained from 20 smokers and 20 non-smokers, matched for age and gender (12 females in each group) and stored at -80°C until required. The mean age of the smoking group was 44.4 [s.d. 6.1] years. The mean age of the non-smoking group was 44.9 [s.d. 6.5] years. Smokers were required to have smoked ≥ 10 cigarettes daily for ≥ 3 years. Those who reported to have consumed no cigarettes in the previous 10 years were considered for inclusion in the non-smoking group. Smoking status was confirmed by serum cotinine analysis (smokers ≥ 50 ng ml^-1 ^cotinine; non-smokers ≥ 10 ng ml^-1 ^cotinine). Exclusion criteria were pregnancy, diabetes, reported history of hypertension, angina, any inflammatory disease such as rheumatoid arthritis or eczema, use of antibiotics within the preceding 2 months, or the current use of anti-inflammatory medication, including NSAID's. Written, informed consent was obtained from each subject, following granting of ethical approval by the local ethics committee.

### Measurement of cotinine

Smoking status was confirmed and tobacco smoke exposure quantified by analysis of serum cotinine, by using a capillary column gas-liquid chromatography technique, as previously described [34]. Cotinine assays were performed blind to self-reported smoking status. The mean coefficient of variation (CV) for analysis of cotinine (over the range 1.0 to 1000 ng ml^-1^) has been determined to be 2.2% [34]. The lower limit of detection for cotinine in this system is 100 pg ml^-1^. Smokers can be reliably differentiated from non-smokers with over 99% confidence using an optimal cut-off value of >13.7 ng ml^-1 ^serum cotinine [35].

### Measurement of IGF-1

IGF-1 concentrations were measured in duplicate by immunoassay (Quantikine IGF-1 Immunoassay, R&D Systems, Minneapolis, MN), using Eschericia coli-expressed recombinant human IGF-1 to generate the standard curve. The mean intra-assay CV, determined by assaying the IGF-1 concentration in three samples in replicates of twenty, is reported by the manufacturer to be 3.0%. The mean inter-assay CV, determined by assaying three samples in forty separate assays, has been determined to be 8.0%. The mean minimal detectable concentration of IGF-1 in this assay is 0.026 ng ml^-1^.

### Measurement of IGFBP-3

IGFBP-3 concentrations were measured in duplicate by immunoassay (Quantikine IGFBP-3 Immunoassay, R&D Systems, Minneapolis, MN), using NSO-expressed recombinant human IGFBP-3 to generate the standard curve. The mean intra-assay CV, determined by the manufacturer by assaying the IGFBP-3 concentration in three samples in replicates of twenty, is reported to be 4.0%. The mean inter-assay CV, determined by assaying three samples in forty separate assays, has been determined to be 6.6%. The mean minimal detectable concentration of IGFBP-3 in this assay is 0.05 ng ml^-1^.

### Measurement of sICAM-1

Soluble ICAM-1 concentrations were measured by ELISA (sICAM-1 Parameter Immunoassay, R&D Systems, Minneapolis, MN) in duplicate, according to the manufacturers instructions, and as previously described [36]. The mean intra-assay CV, determined by assaying the sICAM-1 concentration in three serum samples in replicates of ten, is reported to be 4.4%. The mean inter-assay CV, determined by assaying three serum samples in duplicate in 18 separate assays by four operators, has been determined to be 7.4%. The reported sensitivity of the ELISA is less than 0.35 ng ml^-1^.

### Statistical analysis

Statistical analysis was carried out using STATA 7.0 software (Stata Corp., Texas 77845, USA). Differences between smoking groups were analyzed using a two-group t-test. Relationships between variables were assessed using Spearman correlation.

## Results

The smoking status of all subjects was validated by serum cotinine analysis. The serum cotinine concentrations (mean [s.d.]) in the smoking and non-smoking groups were 251.8 [89.8] and 0.79 ng ml^-1 ^[0.72] (p < 0.001), respectively.

**Figure 1:**
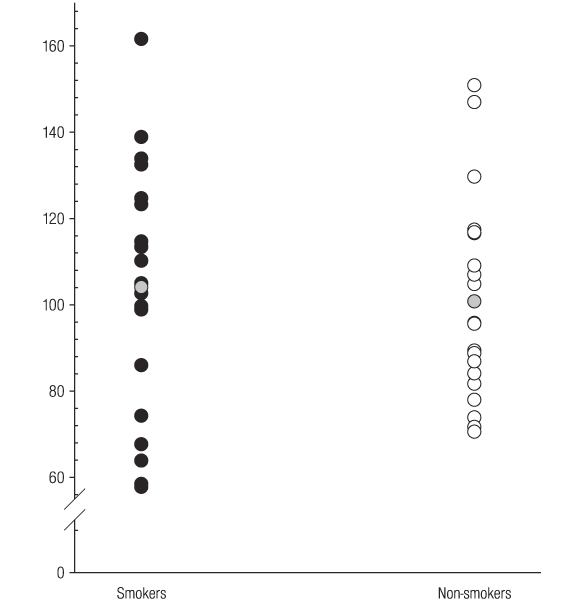
**Serum IGF-1 concentrations ng ml^-1 ^in smokers and non-smokers**. The grey circles represent mean serum concentrations.

Serum IGF-1 levels in individual smokers and non-smokers are shown in Figure 1. There was no significant difference in IGF-1 concentrations between smokers and non-smokers (mean [s.d]; 104 [29] and 101 [24] ng ml^-1^, respectively).

**Figure 2:**
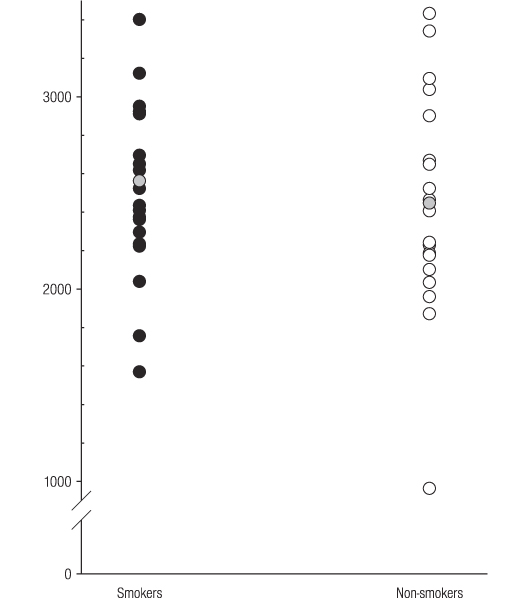
**Serum IGFBP-3 concentrations ng ml^-1 ^in smokers and non-smokers**. The grey circles represent mean serum concentrations.

Serum IGFBP-3 concentrations in individual smokers and non-smokers are presented in Figure 2. No significant difference in IGFBP-3 concentrations measured between smokers and non-smokers (mean [s.d]; 2562 [522] and 2447 [570] ng ml^-1^, respectively) was observed.

Furthermore, there was no significant difference in circulating IGF-1: IGFBP-3 ratios between smokers and non-smokers (mean [s.d]; 0.041 [0.008] and 0.044 [0.017], respectively). Similarly, there was no correlation between serum cotinine and IGF-1 (r = -0.005, p = 0.978) or IGFBP-3 (r = 0.079, p = 0.628) concentrations in smokers.

**Figure 3:**
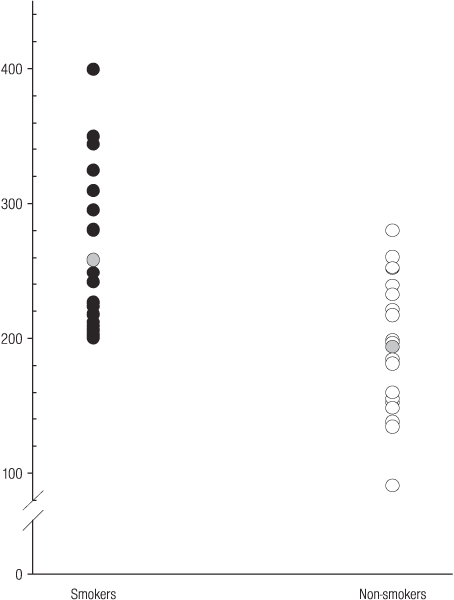
**Serum ICAM-1 concentrations ng ml^-1 ^in smokers and non-smokers**. The grey circles represent mean serum concentrations.

Systemic sICAM-1 concentrations, as expected [32,33], were significantly elevated in smokers, compared to non-smokers (mean [s.d]; 258 [60] and 194 [50] ng ml^-1^, respectively; p = 0.002), as shown in Figure 3.

## Discussion

Previous studies that have addressed the possibility of a direct relationship between tobacco smoking and the systemic load of IGF-1 and/or IGFBP-3 have been inconsistent in their conclusions. Probst-Hensch et al. [29] observed an increase in IGF-1 concentration in smokers, correlating with the number of cigarettes consumed daily, but no difference in circulating IGFBP-3 concentrations between smokers and non-smokers. Kaklamani et al [27] also reported that IGF-1 concentrations are higher in smokers, and positively associated with an index of cumulative smoke exposure (pack-years).

Elsewhere in the medical literature, a negative relationship between IGF-1 and the amount of tobacco smoked has been reported, in men only [31]. In another relevant study, Coutant et al [37] showed that IGF-1 was reduced in cord blood of neonates from smoking mothers compared to neonates from non-smoking mothers, and that this reduction was consistent with their lower birth weight percentile. Others have indicated that circulating IGF-1 levels may be lowered in smokers, compared to non-smokers [23]. Others have found a minimal or no relationship between tobacco smoking and IGF-1 concentrations [19,20,28].

Recently, Renehan et al [30] recognized that both tobacco smoking and changes in the IGF/IGFBP system have been implicated as risk factors for common epithelial cancers. These authors, therefore, examined the relationship between cigarette smoke exposure and serum IGF-1, and IGFBP-3 levels. No significant difference in mean serum IGF-1 between smokers and non-smokers was observed. Mean IGFBP-3 concentrations, on the other hand, were significantly lower in smokers. These results are in agreement with a previous study that reported serum levels of IGFBP-3 to be independently and negatively associated with the number of cigarettes smoked per day or pack-year history of smoking [27]. However, smoking status in the majority of previous studies has been determined by questionnaire, which is known to be unreliable [38]. Furthermore, subjects were often not matched for gender and/or age, both known to influence systemic IGF-1 and IGFBP-3 concentrations.

Thus, with respect to the relationship between tobacco use and IGF-1/IGFBP-3 ratios, the present study is an improvement on previous study designs. Smoking subjects have been matched for age and gender with non-smokers, smoking status validated biochemically, and tobacco smoke exposure quantified by serum cotinine analysis. Furthermore, all subjects in the present study were healthy, with no known confounding disease or condition that may influence IGF-1 or IGFBP-3 concentrations.

Unlike ICAM-1 (Figure 3), no relationship between tobacco smoking and IGF-1 (Figure 1) or IGFBP-3 (Figure 2) was observed. Therefore, tobacco smoking would not appear to be a major confounder of the reported clinical associations between IGF-1, IGFBP-3, or IGF-1/IGFBP-3 ratios and specific disease entities, including certain malignancies and vascular disease.

## Competing interests

The authors declare that they have no competing interests.
